# Inattention in primary school is not good for your future school achievement—A pattern classification study

**DOI:** 10.1371/journal.pone.0188310

**Published:** 2017-11-28

**Authors:** Astri J. Lundervold, Tormod Bøe, Arvid Lundervold

**Affiliations:** 1 Department of Biological and Medical Psychology University of Bergen, 5009 Bergen, Norway; 2 K.G. Jebsen Center for Research on Neuropsychiatric Disorders, University of Bergen, Bergen, Norway; 3 Regional Centre for Child and Youth Mental Health and Child Welfare, Uni Research Health, Bergen, Norway; 4 Neuroinformatics and Image Analysis Laboratory, Neural Networks Research Group, Department of Biomedicine, University of Bergen, Bergen, Norway; Hamamatsu Ika Daigaku, JAPAN

## Abstract

Inattention in childhood is associated with academic problems later in life. The contribution of specific aspects of inattentive behaviour is, however, less known. We investigated feature importance of primary school teachers’ reports on nine aspects of inattentive behaviour, gender and age in predicting future academic achievement. Primary school teachers of *n* = 2491 children (7–9 years) rated nine items reflecting different aspects of inattentive behaviour in 2002. A mean academic achievement score from the previous semester in high school (2012) was available for each youth from an official school register. All scores were at a categorical level. Feature importances were assessed by using multinominal logistic regression, classification and regression trees analysis, and a random forest algorithm. Finally, a comprehensive pattern classification procedure using *k*-fold cross-validation was implemented. Overall, inattention was rated as more severe in boys, who also obtained lower academic achievement scores in high school than girls. Problems related to sustained attention and distractibility were together with age and gender defined as the most important features to predict future achievement scores. Using these four features as input to a collection of classifiers employing *k*-fold cross-validation for prediction of academic achievement level, we obtained classification accuracy, precision and recall that were clearly better than chance levels. Primary school teachers’ reports of problems related to sustained attention and distractibility were identified as the two most important features of inattentive behaviour predicting academic achievement in high school. Identification and follow-up procedures of primary school children showing these characteristics should be prioritised to prevent future academic failure.

## Introduction

Inattention in early childhood has been linked to a wide range of behavioural and social problems [1, 2], including poor academic achievement. This has been shown in several studies of individuals with Attention Deficit Hyperactivity disorder (ADHD, see e.g. [3, 4]), but is also widely documented in studies including community samples [5–11]. In most of these studies, inattention is defined as a sum score across a set of items.

Inattention is, however, a multidimensional concept, where the items reflect impairment of sustained and focused attention, impaired working memory, distractibility, forgetfulness, as well as impaired ability to organise and plan activities and tasks. These aspects of inattention have been described as independent at a biological level [12], but may be extremely difficult to disentangle behaviourally. They rather tend to occur as patterns of behaviour. For example, most children may be distracted by external stimuli in a classroom situation [13], and these distractions will probably be especially hard to handle by a child who has problems maintaining attention and engagement in a task. Thus, it may not be the total inattention score, but rather specific patterns of inattentive behaviour that have the most detrimental effect on the child’s present and future function at school. Identification of important features of inattentive behaviour will therefore be of great importance when developing remediation procedures.

Primary school teachers’ skills are crucial in the work to detect and help a child struggling with inattention. They observe their pupils on a regular basis and in a wide range of situations were inattention tends to have negative effects on performance. At the same time, one should be aware of the risk of biases. Primary school teachers may for example be more tolerant to the behaviour of a child in the lowest class levels, and previous studies have shown that teachers tend to rate girls as less impaired than boys, even when the girls exhibit problematic behaviour in the classroom [14–16]. The child’s gender and age should therefore be taken into account when evaluating teacher ratings of inattentive behaviour.

The aim of the present study was to further investigate the importance of primary school teachers’ reports of inattentive behaviour. To that end, we included data from the Bergen Child Study, where primary school teachers completed a questionnaire including nine items reflecting different aspects of inattentive behaviour when the children were between 7 and 9 years old. About ten years later, when the children had become high school students, academic achievement scores from the official school registry of Norway were available for a subset of the children from the original sample. Described as a key determinant of later occupational career success and adult financial stability [17], there are strong arguments for using academic achievement as an outcome variable. Each of the nine inattention items were rated on a Likert scale with three response alternatives, and the outcome variable, academic achievement, was discretised into three intervals, including an almost equal number of participants in each category. Teacher scores on each of the nine items were used as predictors together with gender and primary school class level (a proxy for age) to answer the following questions: (**1**) which features of inattentive behaviour in primary school represent the strongest predictors of academic achievement in high school? (**2**) how well can the result be generalised to an independent data set?, and (**3**) are gender and the age of the child when evaluated by their primary school teachers of importance to the prediction?

In this context, statistical machine learning approaches were selected according to the following criteria: (**i**) the methods must handle multiple predictors with a small set of response alternatives, and with a small set of outcome categories; (**ii**) the methods should be generic and of interest to other similar data analysis situations and prediction challenges occurring in the behavioural sciences, and (**iii**) the methods should produce results that are easy to interpret at a clinical level. Based on these criteria we selected *multinomial logistic regression* (MLR), *classification and regression trees* (CART), and a *random forest algorithm* (RF) to assess feature importance, and a *k-fold cross-validation procedure* to estimate the classification accuracy, precision, and recall of a model using, in the prediction, the most important features being identified.

## Materials and methods

The data included in the present study are from the Bergen Child Study (BCS), a longitudinal, population-based study on mental health and development. The first wave of the BCS was launched in October 2002, and included the total population of 9,430 children attending second to fourth grade (7-9 years old, born in 1993, 1994 and 1995) in all public, private, and special schools in Bergen. During the initial screening phase, parents and teachers were asked to complete a four-page questionnaire, including, among other scales, a somewhat modified Swanson, Nolan, and Pelham Questionnaire—Fourth Edition (SNAP-IV) [18]. Sample protocols of the first wave have been described in several previous publications from the Bergen Child Study group (e.g., [19–21]).

A fourth and final study-wave was conducted when the youth were between 16 and 19 years old. The sample for this wave included all adolescents born between 1993 and 1995 living in the county of Hordaland (*n* = 10,222). This county includes the city of Bergen, and the BCS sample was thus nested within this Hordaland sample. Academic achievement scores from the previous semester in high school were made available from the official school registry. The BCS was approved by the Regional Committee for Medical and Health Research Ethics (REC), Western Norway (2015/800 Barn i Bergen/ung@hordaland). Parents gave written consent for participation in the first wave of the study. In accordance with the regulations from the REC and Norwegian health authorities, adolescents aged 16 years and older can make decisions regarding their own health (including participation in health studies), and thus gave consent themselves to participate in the fourth wave of the study. Parents/guardians have the right to be informed, and in the current study, all parents/guardians received written information about the study in advance. More information about the project is given at the BCS homepage: http://uni.no/en/bergen-child-study.

### The sample

The sample included *n* = 2491 participants (*n* = 1192 boys). All participants were rated by their primary school teachers on all selected SNAP-IV items when they were 7 to 9 years old (primary school class levels 2, 3, or 4), and information about gender and academic achievement score were available when they attended high school (16 to 19 years old). Within this sample, the percentages of children attending 2^nd^, 3^rd^ and 4^th^ primary school class levels when evaluated by their teachers were 42.3%, 34.4% and 23.4%, respectively.

### Teacher reports

*Inattention* items were selected from the SNAP-IV [18], a scale which describes problems used to define the inattentive symptoms of the Attention Deficit Hyperactivity Disorder (ADHD) according to the Diagnostic and Statistical Manual of Mental Disorders (DSM-5) [22]. The original SNAP-IV uses four levels to evaluate each item, whereas in our study, the teachers evaluated each item on a 3-level Likert-type scale (“not true”, “somewhat true”, or “certainly true”) in order to follow the response pattern of the remaining scales included in the first wave of the BCS questionnaire. Each answer was assigned a value 0, 1, or 2. The nine inattention items from SNAP-IV are listed in Table 1.

**Table 1:** SNAP items, scored as “not true” (0), “somewhat true” (1), and “certainly true” (2).

*SNAP1*:	Often fails to give close attention to details or makes careless mistakes in schoolwork, work, or other activities
*SNAP2*:	Often has difficulty sustaining attention in tasks or play activities
*SNAP3*:	Often does not seem to listen when spoken to directly
*SNAP4*:	Often does not follow through on instructions and fails to finish schoolwork, chores, or duties
*SNAP5*:	Often has difficulty organising tasks and activities
*SNAP6*:	Often avoids, dislikes, or is reluctant to engage in tasks that require sustained mental effort
*SNAP7*:	Often loses things necessary for tasks or activities (e.g., toys, school assignments, pencils, books, or tools)
*SNAP8*:	Often is distracted by extraneous stimuli
*SNAP9*:	Often is forgetful in daily activities

The percentages of children scored within the three response categories are given in Table 2, confirming that the frequency of girls reported with a “not true” response was significantly higher than in boys.

**Table 2:** Percentage of children obtaining a given response from their teachers on each inattention item (SNAP-IV).

	“Not true”			“Somewhat true”			“Certainly true”		
*n* =	All	Girls	Boys	All	Girls	Boys	All	Girls	Boys
	2167	1186	981	278	97	181	46	6	30
*SNAP1*	87.0	91.3	82.3**	11.2	7.5	15.2	1.8	1.2	2.5
*SNAP2*	88.5	94.1	82.5**	9.5	5.5	13.8	2.0	0.5	3.7
*SNAP3*	92.0	96.7	86.9**	7.4	3.1	12.1	0.6	0.2	1.0
*SNAP4*	92.5	96.2	88.4**	6.8	3.6	10.4	0.7	0.2	1.1
*SNAP5*	91.5	96.1	86.5**	7.3	3.5	11.4	1.2	0.5	2.1
*SNAP6*	91.7	96.3	86.7**	7.0	3.3	11.1	1.3	0.4	2.3
*SNAP7*	96.5	98.5	94.4**	3.0	1.2	4.9	0.5	0.3	0.7
*SNAP8*	75.2	84.4	65.1**	21.0	14.1	28.4	3.9	1.5	6.5
*SNAP9*	89.5	93.4	85.2**	9.3	6.2	12.7	1.2	0.4	2.1

*Note*: The overall number of children (*n*) with a given response from their teachers are given in the third row.

**: *p* value <.001 according to a chi-square test comparing a “not true” report in boys and girls.

### Academic achievement

Academic achievement scores were provided by the official registers from the Hordaland County. In Norway, secondary schools use a scale spanning from 1 to 6, with 6 being the highest grade (outstanding competence), 2 the lowest passing grade (low level of competence), and 1 being a *fail*. The scores included in the present study were the mean value of the grades during the previous semester, comprising all school subjects except for physical education. The mean score for girls was statistically significant higher (*μ* = 4.11 (SD = 0.72)) than for boys (*μ* = 3.90 (SD = 0.72), *p* < .001). For the present study, the academic achievement scores were categorised into three levels, calculated to generate groups with a similar number of participants (see details below).

### Statistical analysis

**Fig 1:**
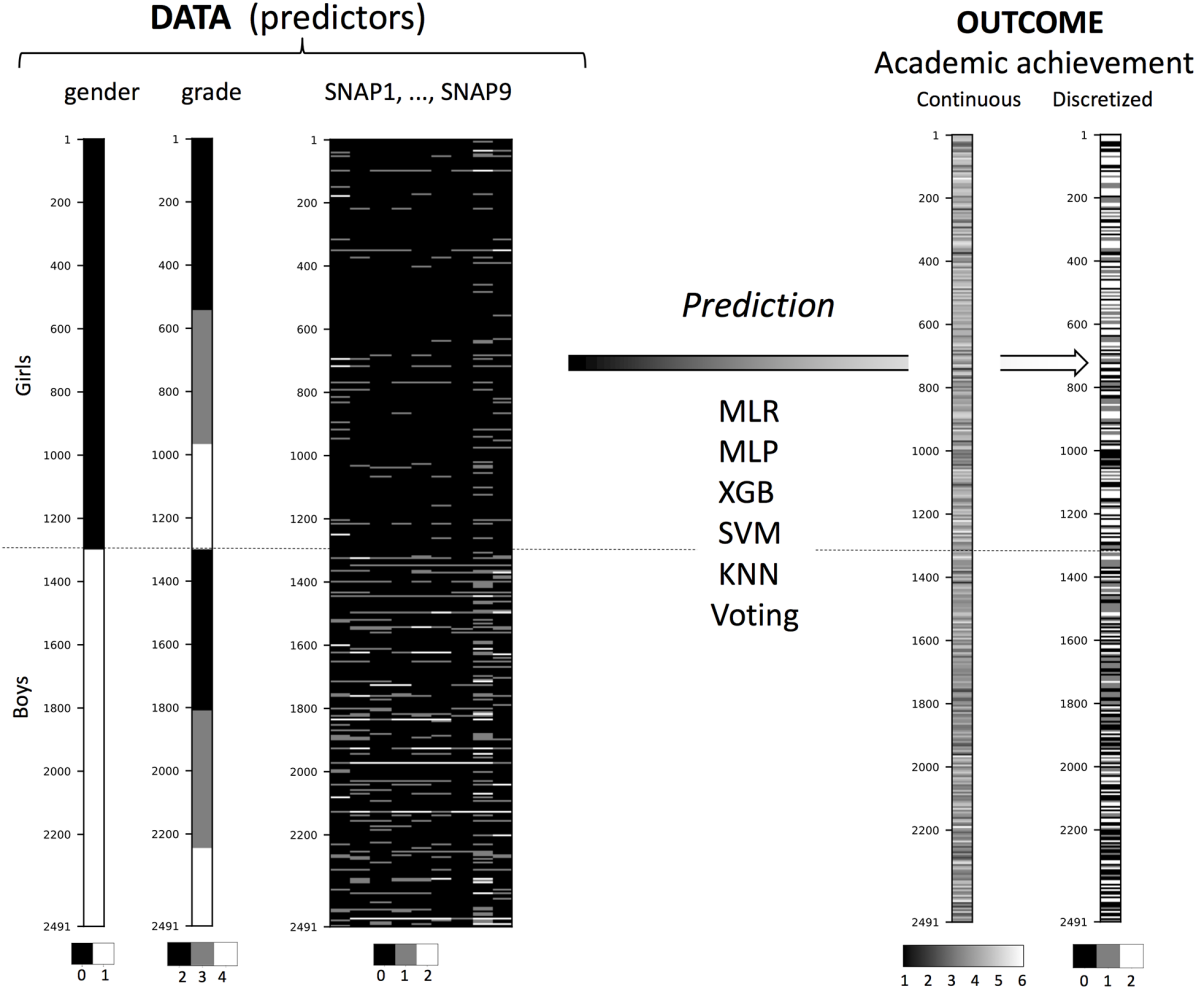
The predictor data (explanatory variables), the academic achievement outcome, and the types of classification analyses being performed. Data values are represented as grey level heat maps. MLR = multinomial logistic regression, MLP = multi-layer perceptron, XGB = extreme gradient boost, SVM = support vector machine, KNN = k-nearest neighbor, Voting = voting (argmax) classifier across MLR, …, KNN.

**Fig 2:**
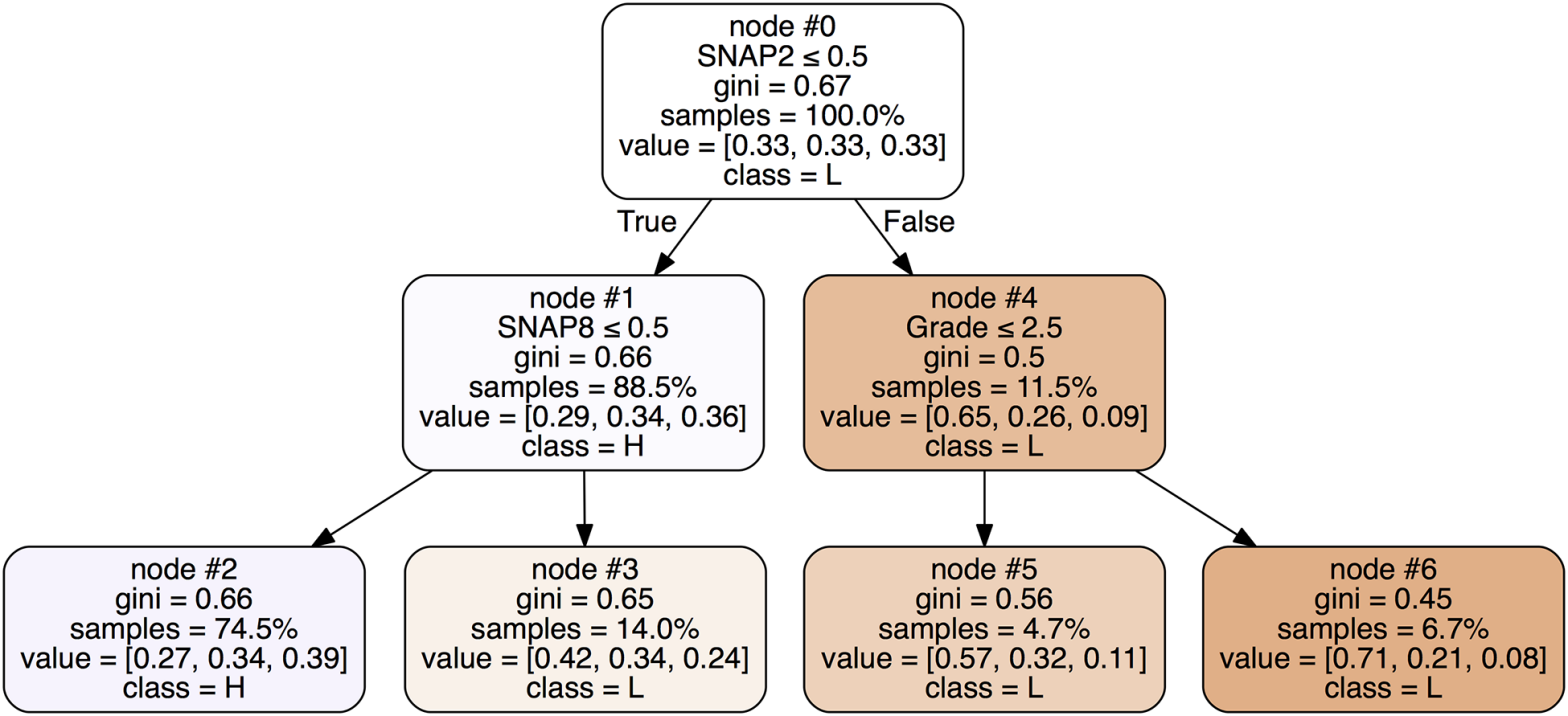
Fitted classification tree (CART analysis), including the predictor variables SNAP-IV items 1 to 9 (0 = “not true”; 1 = “somewhat true”; 2 = “certainly true”); gender (0 = *girl*; 1 = *boy*); grade (primary school class level 2, 3, 4) and the academic achievement outcome (L = *low*, M = *medium*, H = *high*). The percentage in each node box denote the percentage of samples routed to that particular node—where the root node will contain 100% of the samples, and a leaf node will contain the least number of samples along a rooted path in the decision tree. The node numbers are given on top of each node box. For each split decision, *True* denotes that the corresponding statement is true and then pointing to the left child node (that is either a new internal decision node or a final leaf node), and *False* denotes that the corresponding statement is false and then pointing to the right child node (that is either a new internal decision node or a final leaf node).

**Fig 3:**
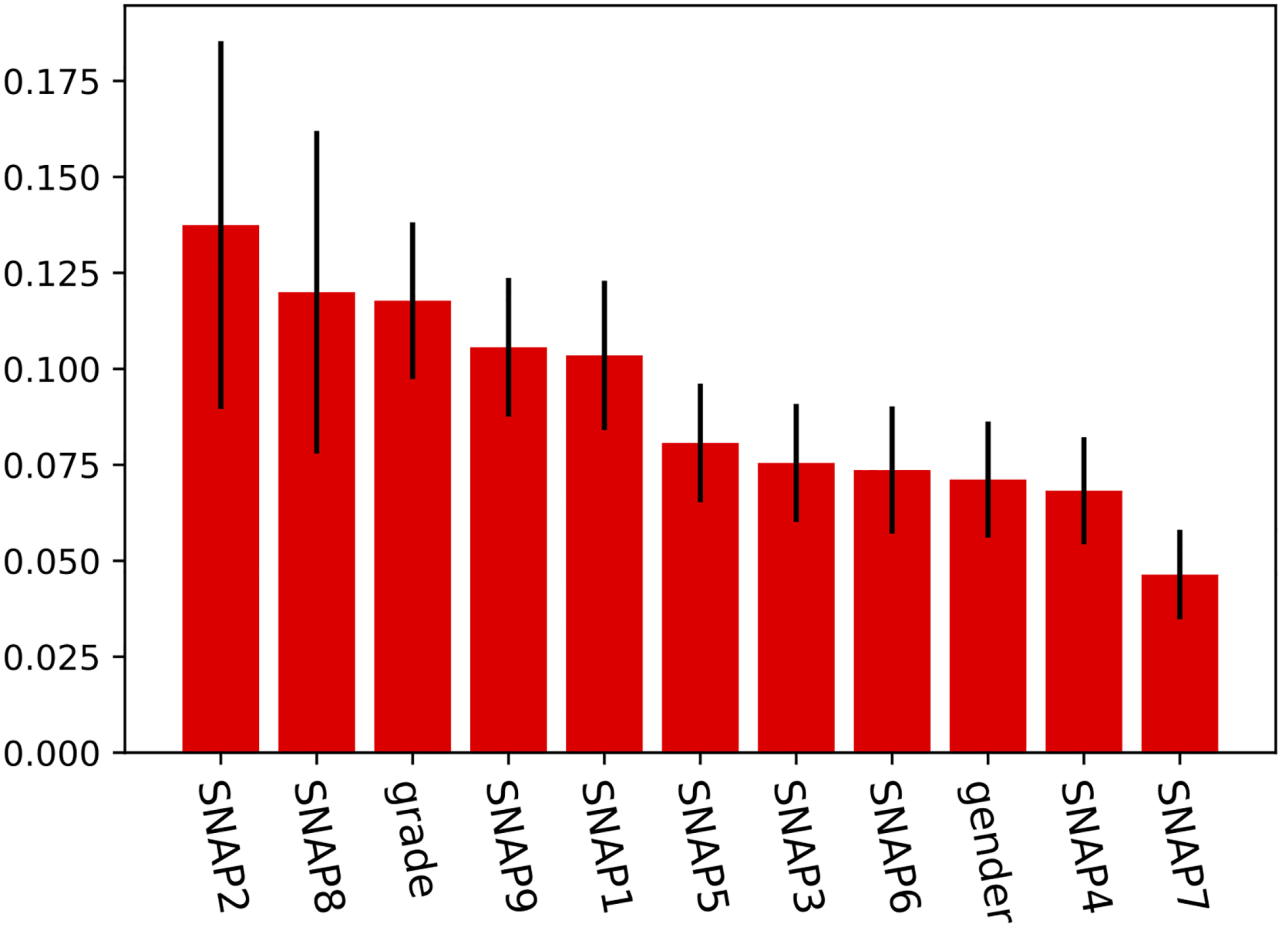
The ranked importance of *SNAP2*, *SNAP8*, *gender* and *grade* (primary school class level) in predicting academic achievement outcome according to the random forest algorithm.

The data analysis was divided into three parts: (**a**) data preparation, including discretising the average academic achievement into three levels, (**b**) casting the data analysis problem into a machine learning classification task assessing feature importances using both a multinomial logistic regression (MLR), classification and regression trees (CART), and a random forest (RF) algorithm, and (**c**) a pattern classification procedure using *k*-fold cross-validation with five different linear and nonlinear classifiers (MLR, MLP, XGB, SVM, KNN, each described below) and incorporation of a voting classifier across these five. All these steps were implemented in Jupyter notebooks using Python (3.5.4), Numpy (1.12), Pandas (0.20), Statsmodels (0.8), XGBoost (0.6), Scikit-learn (0.19), rpy2 (2.8.5) and Matplotlib (2.0) for producing Figs 1–3. Our Jupyter notebook for computing feature importances and classification with *k*-fold cross-validation will be available on GitHub (https://github.com/arvidl/inattention-populationsample).

#### (a) Data preparation and explorative data analysis

The original data, provided to us as a SPSS-file, were imported into the Jupyter notebook environment via rpy2 and the r-foreign packages. For the analysis we used the sample of *n* = 2491 children having complete data on the 11 predictor variables and mean academic achievement as outcome variable, cfr. Fig 1.

For classification purposes, the average academic achievement scores (*ave*) were discretised into three intervals (level of academic achievement) using Pandas qcut(), to include about the same number of participants in each of the categories: *low* (*ave* ∈ [1.000 − 3.714〉, *n* = 834), *medium* (*ave* ∈ [3.714 − 4.375〉, *n* = 831), and *high* (*ave* [4.375 − 6.000], *n* = 826). The distribution across the three levels—from *low* to *high*—was 40.3%, 33.3% and 26.4% for boys, and 27.3%, 33.4% and 39.3% for girls, confirming the overall higher academic scores achieved by the girls.

Depiction of the complete dataset is given in Fig 1, using gray scale heatmap columns for the *n* = 2491 participants comprising the predictor variables *gender*, *grade*, *SNAP1*, …, *SNAP9*, and the outcome variable *academic achievement*. In Fig 1 we have also listed the six classifiers being used for prediction in a *k*-fold cross validation scheme. The observations above the horizontal dotted line represent girls and below the dotted line are the boys.

#### (b) Assessment of feature importance

To assess feature importances of the 11 candidate variables for predicting *low*, *medium*, and *high* academic achievement in the whole cohort, we performed three types of analyses: (**i**) Multinomial logistic regression with consideration of each parameter, i.e. the magnitude of its coefficient, the standard error of the corresponding parameter, and the odds ratio, (**ii**) a CART analysis with assessment of the top important decision nodes, and (**iii**) a random forest classification using a forest of 10000 trees (“weak learners”) and ordering of features importance according to the ‘gini’ information criterion.

#### Multinomial logistic regression model (MLR)

The multinomial logistic regression analysis included the following set of variables on a nominal level: the three levels of academic achievement scores as outcome variable, and *gender*, primary school class level (*grade*), and teacher reports on the nine inattention items *SNAP1*,…,*SNAP9* as predictors. Generally, the multinomial logistic regression model relates a set of explanatory variables *x*_1_, …, *x*_*p*_ to a set of log-odds, log(*π*_2_/*π*_1_), … log(*π*_*J*_/*π*_1_) according to

$$\begin{array}{c} \log\left(\pi_{j}/\pi_{1}\right)=\beta_{j0}+\beta_{j1}x_{1}+\cdots +\beta_{jp}x_{p} \end{array}$$ (1)

for *j* = 2, …, *J*. Here, *j* = 1 represents the base level category, *π*_*j*_ = *P*(academic achievement level = *j*), *π*_*j*_/*π*_*j*′_ denotes the odds of category *j* relative to *j*′ (i.e. odds ratio, OR), and $\sum_{j=1}^{J}\pi_{j}=1$ (see e.g. [23] for details). In our case, we let the base level category *j* = 1 be the *low* mean academic achievement, meaning that the *low* was compared separately to the *medium* and *high* categories. For computations we used mnlogit() from the statsmodels.formula.api.

#### Classification trees (CART)

The *SNAP1*,…,*SNAP9* items were included together with demographics (*gender* and primary school class level (*grade*)) as predictor variables in a CART analysis [24] used to predict level of academic achievement score {*low*, *medium*, *high*}. In brief, the *root* of the classification tree is the top node and input patterns are passed down the tree such that decisions are made at each node until a terminal node (a *leaf*) is reached. At each non-terminal node a question is posed on which a binary split is made such that the “child” nodes are on average “purer” than their “parent”. A measure of “impurity” is low (i.e. close to 0) if the probability of the occurrence of a class at a given node for all subsets of patterns reaching that node is concentrated on that class. The “impurity” is maximal (i.e. close to 1) if the class probabilities at that node is uniform.

In our analysis we used the DecisionTreeClassifier() from sklearn.tree with impurity *criterion = ‘gini’* and *max_depth = 2* for growing the classification tree.

#### Random forest ensemble learning (RF)

Random forest (RF) is an ensemble learning method for classification that constructs a multitude of decision trees at training time and output the mode class among the generated classes. The RF algorithm involves the construction of *n* trees and ensures that each tree uses a different set of data (bootstrapping) and a different set of variables (“feature bagging”) at each candidate split. Thus, RF is less prone to overfitting compared to CART, and will therefore produce more generalisable results [24]. Moreover, the order of decisions in the hierarchies of trees will reflect the importance of the corresponding feature variables being involved. In our setting, the variables *gender*, *grade*, and the *SNAP1*,…,*SNAP9* items were included as predictors of the outcome level of academic achievement: *low* (*L*), *medium* (*M*), or *high* (*H*). In the analysis we used the RandomForestClassifier() from sklearn.ensemble with impurity *criterion = ‘gini’*, *n_estimators = 10000*, *bootstrap = True*, *max_features = None*, and *max_depth = None*. After fitting the forest with the 2491 × 11 predictor matrix *X* and academic achievement outcome *y* ∈ {*L*, *M*, *H*}, i.e. forest.fit(X,y), the Scikit-learn RF algorithm enables the calculation of forest.feature_importances_.

#### (c) Prediction using *k*-fold cross-validation

From the feature importance step, the top ranked predictors of academic achievement scores were selected for a comprehensive classification study using *k*-fold cross-validation to assess prediction properties (accuracy, precision, and recall). In this procedure we used both linear classifiers (multinomial logistic regression = MLR) and non-linear classifiers (multi-layer perceptron = MLP, extreme gradient boosting = XGB, a radial basis function kernel support vector machine = SVM, and k-nearest neighbours = KNN).

For the *k*-fold cross-validation we used StratifiedKFold() from sklearn.model_selection with *n_splits = 10* and *shuffle = True*, where the folds (splits) are made by preserving the percentage of samples for each class. For a given fold 1, …, *k* = 10, fixed pairs of (*X_train, y_train*) and (*X_test, y_test*) datasets were provided for each of the six classifiers using the Pipeline mechanism in Scikit-learn, and the feature vectors were standardized to zero mean and unit variance using the StandardScaler() from sklearn.preprocessing. For the individual classifications we used the LogisticRegression() with *solver = ‘saga’* and *multi_class = ‘multinomial’* from sklearn.linear_model; the MLPClassifier() with *hidden_layer_size = 3*, *activation = ‘relu’*, and *solver = ‘adam’* from sklearn.neural_network; the XGBClassifier with *n_estimators = 1000* and *max_depth = 3* from xgboost; the SVC() with *C = 1.0*, *kernel = ‘rbf’*, and *degree = 3* from sklearn.svm; and the KNeighborsClassifier() with *n_neighbors = 5* and *metric = ‘minkowski’* from sklearn.neighbors. To obtain an ensemble voting across the five different classifier we used the VotingClassifier() with *estimators = [MLR, MLP, XGB, SVM, KNN]* and *voting = ‘soft’* from sklearn.ensemble, predicting the class label based on the argmax of the sums of the predicted probabilities.

For the performance assessment on each (*X_test, y_test*) we used *accuracy_score* (the ratio of correct classifications), *precision_score* (the ratio *tp*/(*tp* + *fp*), where *tp* is the number of true positives and *fp* number of false positives), *recall_score* (sensitivity, the ratio *tp*/(*tp* + *fn*) where *fn* is the number of false negatives), and *f1_score* (harmonic mean of the precision and recall) from sklearn.metrics. Finally we computed the mean and standard deviations of these classifier-specific performance measures across the *k* folds.

## Results

We first report the results from the analysis of feature importance, then the prediction results from *k*-fold cross validation using the six different classifiers.

### Assessment of feature importance

#### Multinomial logistic regression model (MLR)

Performing MLR on the complete dataset, *gender* significantly predicted whether a child obtained a *low* rather than a *high* academic achievement score in high school (OR = 0.60, *p* < .002) as well as a *low* rather than a *medium* score (OR = 0.79, *p* < 0.001). This shows that the boys (1) were overall more likely to obtain a *low* academic achievement score in high school than the girls (0) (Table 3).

**Table 3:** Multinomial logistic regression model.

Ref. category:						95%CI		
Low score	Variable	Estimate	SE	z	P>|z|	OR	[0.025	0975]
Medium score	intercept	0.74	0.20	3.72	<0.001	2.09	0.35	1.12
	*gender*	-0.24	0.11	-2.29	0.022	0.79	-0.44	-0.03
	*grade*	-0.15	0.07	-2.51	0.023	0.86	0.29	-0.04
	*SNAP1*	-0.01	0.14	-0.04	0.970	0.99	-0.27	0.26
	*SNAP2*	-0.62	0.19	-3.24	0.001	0.54	-1.00	-0.25
	*SNAP3*	0.06	0.19	0.31	0.757	1.06	-0.32	0.44
	*SNAP4*	0.21	0.23	0.89	0.374	1.23	-0.25	0.67
	*SNAP*5	0.01	0.22	0.04	0.970	1.01	-0.42	0.44
	*SNAP6*	-0.19	0.20	-0.94	0.350	0.83	-0.58	0.21
	*SNAP7*	0.16	0.26	0.60	0.550	1.17	-0.35	0.67
	*SNAP8*	-0.28	0.13	-2.27	0.023	0.75	-0.53	-0.04
	*SNAP9*	-0.09	0.16	-0.55	0.581	0.92	-0.40	0.23
High score	intercept	0.78	0.20	3,88	<0.001	2.19	0.39	1.18
	*gender*	-0.51	0.11	-4.83	<0.001	0.60	-0.72	-0.30
	*grade*	-0.07	0.07	-1.05	0.292	0.93	-0.20	0.06
	*SNAP1*	-0.50	0.18	-2.80	0.005	0.61	-0.85	-0.15
	*SNAP2*	-0.93	0.26	-3.55	<0.001	0.40	-1.44	-0.42
	*SNAP3*	-0.09	0.25	-0.38	0.704	0.91	-0.57	0.39
	*SNAP4*	0.18	0.32	0.57	0.567	1.20	0.44	0.80
	*SNAP5*	0.46	0.27	1.69	0.091	1.58	-0.07	0.99
	*SNAP6*	-0.74	0.29	-2.58	0.010	0.48	-1.30	-0.18
	*SNAP7*	-0.28	0.43	-0.66	0.511	0.75	1.13	0.56
	*SNAP8*	-0.61	0.15	-4.16	<0.001	0.55	-0.89	-0.32
	*SNAP9*	-0.14	0.19	-0.74	0.462	0.87	-0.51	0.23

Reference group = low academic achievement. OR = Odds ratio.

Two of the teacher reported inattention items significantly predicted a *low* rather than a *medium* academic achievement score. The strongest effect was found for an item reflecting problems related to sustained attention, *SNAP2* (*p* = 0.001). An odds ratio of .54 tells us that for each unit change in the score given by the teacher, the child was almost two times less likely to obtain a *medium* compared to a *low* academic achievement score (1/.54 = 1.9). The second item reflects distractibility, *SNAP8* (*p* = 0.02, OR = 0.75), leaving the child with a somewhat increased odds (1.3) of obtaining a *low* score.

Predictions from the two inattention items were even stronger when comparing *low* to *high* academic achievement scores, with the highest estimate on *SNAP2* (*p* < 0.001) followed by *SNAP8* (*p* < 0.001). The odds ratios show that the child was 2.5 times more likely to obtain a *low* than *high* score in high school for each more severe step in problems reported on *SNAP2* (OR = 0.40) and 1.8 times more likely for each step on *SNAP8* (OR = 0.55). The prediction of *low* rather than *high* academic achievement score was also significant for two other items reflecting problems related to sustained attention, *SNAP1* (*p* = 0.05) and *SNAP6* (*p* = 0.001). With ORs of 0.61 and 0.48, the increase was around twofold (1.6 and 2, 1, respectively). *SNAP5* (*p* = 0.009) gave a more surprising result, with a higher likelihood to obtain a high academic achievement level if reported with disorganised behaviour by your primary school teacher.

To sum up the results from the MLR, inattentive behaviour associated with problems related to sustained attention and distractibility predicted *low* rather than *medium* or *high* academic achievement levels in high school, with an overall higher odds-ratio in boys than in girls (Table 3).

#### Classification trees (CART)

The CART analysis, using maximum depth = 2, generated four terminal nodes (Fig 2).

The first and most important split (the top node #0) was on *SNAP2*, assessing problems related to sustained attention. The “False” branch at this node, i.e. teachers reporting “somewhat true” or “certainly true” on this item, arriving at node #4 (11.5% of the sample), were mainly associated with a *low* academic achievement score in high school. In this subsample, primary school class level (*grade*) did matter. A higher portion of those those with “somewhat true” or “certainly true” reports on *SNAP2* in the 3^rd^ and 4^th^ grades (node #6) obtained *lower* academic scores than those in the 2^nd^ grade (node #5), 71% and 57%, respectively.

If primary school teachers reported “not true” on *SNAP2*, then the reports on problems related to distractibility (*SNAP8*) was important for prediction, i.e. node #1 comprising 88.5% of the sample. Reporting “somewhat true” or “certainly true” on *SNAP8*, i.e. the “false” branch from node #1 to node #3, led to the highest percentages towards a *low* academic achievement score (42%), while a “not true” report (node #2, 74.5% of the sample) was associated with the highest percentages towards the *high* score (39%).

To sum up the results from the CART analysis, problems related to sustained attention (*SNAP2*) or distractibility (*SNAP8*) were important predictors of *low* school academic achievement scores in 25.5% (nodes #3 and #4) of the children. Primary school class *grade* did also matter, with a higher percentage of children obtaining a *low* score when assessments were done at 3^rd^ or 4^th^ grade.

#### Random forest ensemble learning (RF)

The random forest algorithm with 10000 trees further explored feature importances in the cohort. Fig 3 shows the ranked importance of the 11 predictor variables, confirming the main findings from the MLR and CART analyses, where to top three most important features were *SNAP2* > *SNAP8* > *grade*.

### Prediction using *k*-fold cross-validation

The cross-validation procedure was performed separately for the three top features selected by the RF analysis. Gender was included due to its effect upon both the SNAP-IV items and the academic achievement score, and the statistically significant effect revealed by the MLR. Table 4 shows the results from the selected classifiers and the overall voting on the accuracy, precision and recall measures. All values were above chance level (> 33%) for the three categories of academic achievement scores.

**Table 4:** Classification performance using *k*-fold cross-validation (*k* = 10).

	MLR	MLP	XGB	SVM	KNN	Voting
*Accuracy*	0.43 (0.03)	0.43 (0.03)	0.44 (0.02)	0.44 (0.02)	0.39 (0.03)	0.42 (0.02)
*Precision*	0.43 (0.04)	0.42 (0.04)	0.45 (0.03)	0.45 (0.04)	0.38 (0.03)	0.42 (0.03)
*Recall*	0.43 (0.03)	0.43 (0.03)	0.44 (0.02)	0.44 (0.02)	0.39 (0.03)	0.42 (0.02)

*Note*: MLR = multinomial logistic regression; MLP = multi-layer perceptron; XGB = extreme gradient boosting; SVN = support vector machine; KNN = k-nearest neighbour; Voting = voting classifier across MLR, MLP, XGB, SVN, and KNN.

## Discussion

### Summary of results

The present study asked if specific features of inattentive behaviour in primary school—as reported by teachers—act as predictors of academic achievement in high school. Different types of multivariate analyses were used to handle the set of categorical variables. Overall, items reflecting problems related to sustained attention and distractibility were selected as the two most important features of inattention in predicting the achievement score. Gender and a proxy for age (primary school class level) were added as important features by the MLR analysis. The CART analysis showed that as many as 25.5% of the children were reported with either of the two inattention problems, and that these children had a high risk of obtaining a low academic achievement score. Age when assessed by their primary school teachers was of some importance, in that the chance of obtaining a low achievement score was somewhat lower when reported with problems in the 2^nd^ than in higher grades (3^rd^ and 4^th^ grades). Age and the items reflecting sustained attention and distractibility were also identified with the highest importance by the RF analysis, suggesting that these results are expected to generalise to other samples. This was confirmed by the *k*-fold cross-validation analyses.

### Early predictors of academic achievement in high school

The present results showed that problems related to sustained attention and distractibility in primary school are important drivers of poor academic performance in high school. By this, the results partly overlapped with findings previously reported in a study by Holmberg et al. [9], where teacher reports of failure to finish a task were found to be one of the main factors explaining academic outcome. Our study add to this by revealing the importance of problems related to distractibility. The MLR analysis showed that this problem was associated with an almost two-fold increase in OR of an achievement score in the lower than higher end of the scale. Its importance as a predictor of poor achievement scores was also supported by the CART analysis, with the strongest effect when reported as a problem in the 3^rd^ and 4^th^ grades. In a class situation, the relation between the two is obvious. A child with the ability to stay focused on a task over a longer period of time is expected to be less disturbed by habits and cues in the environment than a child with poor vigilance. This enables the child to obtain the basic skills and knowledge that are of importance to the academic achievement scores as the curriculum becomes more complex at higher grade levels.

Inclusion of information about nine aspects of inattentive behaviour separately in the statistical analyses revealed their relative importance to high school academic performance. Most previous studies have defined inattention as a sum-score from reports of problems reflecting a range of different behaviours. A significant relation between such a sum score and academic achievement was shown in one of our previous studies, including subsamples from BCS and the Berkeley Girls with ADHD Longitudinal Study (BGALS). Inattention was found to be significant across these two culturally and diagnostically diverse groups, and the effect was over and above the effect of demographics and intellectual function [25]. The present results indicate that the effect on academic achievement is driven by a few features defined within the full inattention score.

The cross-diagnostically effect of inattention was confirmed by the present study. Although inattention is one of the core symptoms of ADHD, the importance of inattentive behaviour in explaining future academic success is definitely not restricted to a diagnostic category; the present study documented this effect in a population-based sample. However, although a high proportion of children obtaining a low academic achievement score were reported as inattentive by their primary school teachers, the cross-validation analyses revealed that more information about the child is needed to obtain an improved validation of the prediction. This probably reflects both the instability of inattentive behaviour and the large number of co-existing and new challenges influencing a child through childhood and adolescence. Previous studies have for example shown the importance of socio-economic factors in general (e.g., [26]), with some cultural differences regarding the importance of its subcomponents [27] and consequences [28]. Further studies should thus include a larger number of predictors and a more diverse sample than in the present study.

Taken together, the present results should inspire assessment and treatment efforts in primary school children vulnerable to distractibility and with problems to sustain their attention in school-related work. The close relationship between inattentive behaviour and cognitive function [29, 30] has lead to increased popularity of presenting cognitive training programs to school children with ADHD (see e.g., [31, 32]). A sole focus on cognitive training of the child is, however, not expected to lead to successful alleviation of the inattentive behaviour described in the present paper. This was supported by the results from the meta-analysis presented by Cortese and collaborators [33], showing that cognitive training procedures had limited effects on ADHD symptoms. Positive contributions from parents and teachers seem to be essential (see e.g., [34]). Whereas parent-focused training produces improvements in negative parenting and impairment at home, incorporation of child skill training and teacher consultation may be necessary to produce improvements at school [35].

Gender turned out to be another important predictor. Girls were reported by their primary school teachers to have less inattention symptoms and to obtain higher academic achievement in high school than boys. Although gender was identified as one of the main predictors of academic achievement scores in the feature extraction by the MLR analysis, it was not selected among the top features of importance by the the CART and RF analyses. Further gender balanced longitudinal studies of functional outcomes of early inattentive behaviour are warranted.

### Strengths and limitations

The large population-based sample of high school students followed from childhood, inclusion of a standardised questionnaire assessing inattention, and inclusion of academic achievement scores from official National registers are main strengths of the present study. Another strength is the inclusion of several statistical methods to assess patterns in the data and to perform predictions—the use of the MLR, CART, and RF algorithms to assess feature importances and select features, and the comprehensive *k*-fold cross-validation procedure. We believe that the relevance of the present analytic approach is not restricted to the topic of the present study, in that questionnaire data with a few response categories are commonly used in psychological research.

In spite of the strengths and the importance of the present study, several limitations must be mentioned. Inclusion of very few features when predicting an outcome about 10 years ahead, is an obvious limitation of the present study. A stronger model could have been obtained by including results from a psychometric test assessing vigilance and distractibility, similar to the one developed by Cassuto et al. [36], or a more ecological valid virtual reality test as the one described by Pelham et al. [37]. Inclusion of teacher reports only may also be considered as a limitation. Furthermore, stronger conclusions could have been obtained by including information from repeated inattention reports to understand the trajectory from early symptoms of inattention to function in adolescence and adulthood. The importance of the latter was demonstrated in a study by Pingault and collaborators [7], showing that increase in symptoms of inattention during childhood really matters when it comes to school graduation failure. Such studies are important and should include analysis of behavioural patterns, because a specific pattern of vigilance and distraction was suggested by the present study. Finally, academic achievement level did not reflect overall high school achievement, in that it was operationalised as the mean of grades for one semester only.
